# Acute macular neuroretinopathy following Moderna COVID-19 vaccination

**DOI:** 10.1186/s12348-023-00354-1

**Published:** 2023-06-29

**Authors:** Olena Protsyk, Roberto Gallego-Pinazo, Rosa Dolz-Marco

**Affiliations:** 1 Department of Ophthalmology, Jaen University Hospital, Av. Del Ejército Español 10, Jaen, 23007 Spain; 2 Unit of Macula, Oftalvist Clinic, Valencia, Spain

**Keywords:** Acute macular neuroretinopathy, COVID-19, Moderna mRNA vaccine

## Abstract

**Purpose:**

To describe the occurrence of an acute macular neuroretinopathy (AMN) after administration of a Moderna COVID-19 Vaccine.

**Methods:**

Case report.

**Results:**

A 23-year-old female presented bilateral visual loss one week after the first dose of COVID-19 vaccine. Fundus examination revealed the classic wedge-shaped lesions with petaloid configuration around both foveas. Hypo-reflective macular lesions are evident in the near-infrared reflectance image. The spectral-domain optical coherence tomography reveled hyperreflectivity of the outer nuclear and plexiform layers, attenuation of the ellipsoid zone and disruption of interdigitation zone corresponding to the lesions.

**Conclusions:**

Despite the large number of doses of COVID-19 vaccines administered worldwide, there are not many reported cases of AMN. Most of them occurred after viral vector vaccines. Described here is one of the few cases that observed a time period of several days after receiving the Moderna messenger RNA vaccine. It is not possible to establish causality although this suggests an inflammatory or autoimmune response to the vaccine.

## Introduction

Acute macular neuroretinopathy (AMN) is a rare pathology of the retina that occurs mostly in young women. It is frequently associated with fever, flu-like symptoms and contraceptive use [1]. During the COVID-19 pandemic there has been increased interest in the condition because of an observed increase in incidence and also because it is the most commonly reported retinal adverse event following COVID-19 vaccination [2, 3].

We present a case of AMN after Moderna COVID-19 Vaccine (ModernaTX, Inc., Cambridge, MA, USA). In addition to the interest of adverse events of COVID vaccination, these cases may help to understand the pathogenesis of AMN.

## Case description

**Fig. 1:**
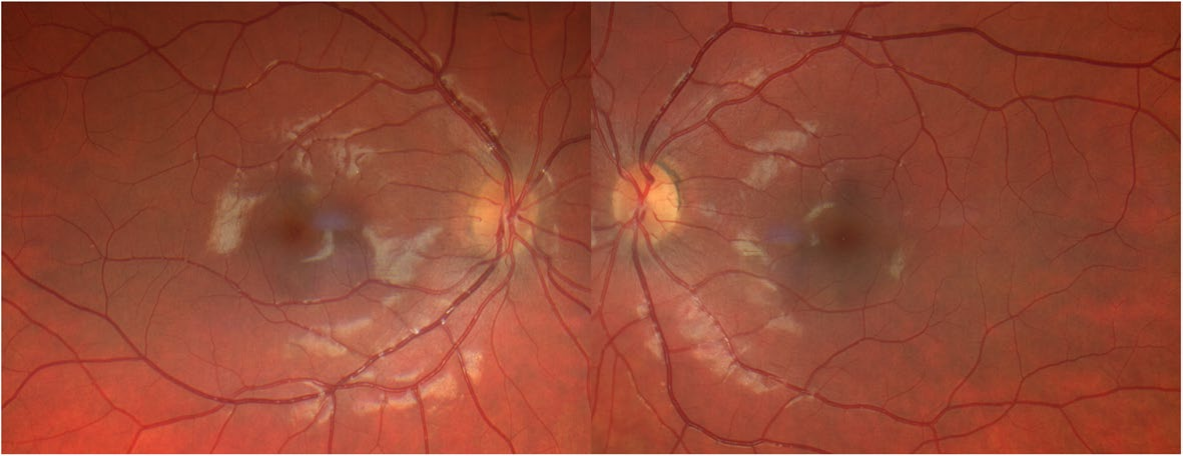
Fundus photography of both eyes. *Left*: Color fundus photo of the right eye shows subtle wedge-shaped lesions around the fovea. *Right*: Color fundus photo of the left eye with subtle wedge-shaped lesions mainly affecting the lower perifoveal area

**Fig. 2:**
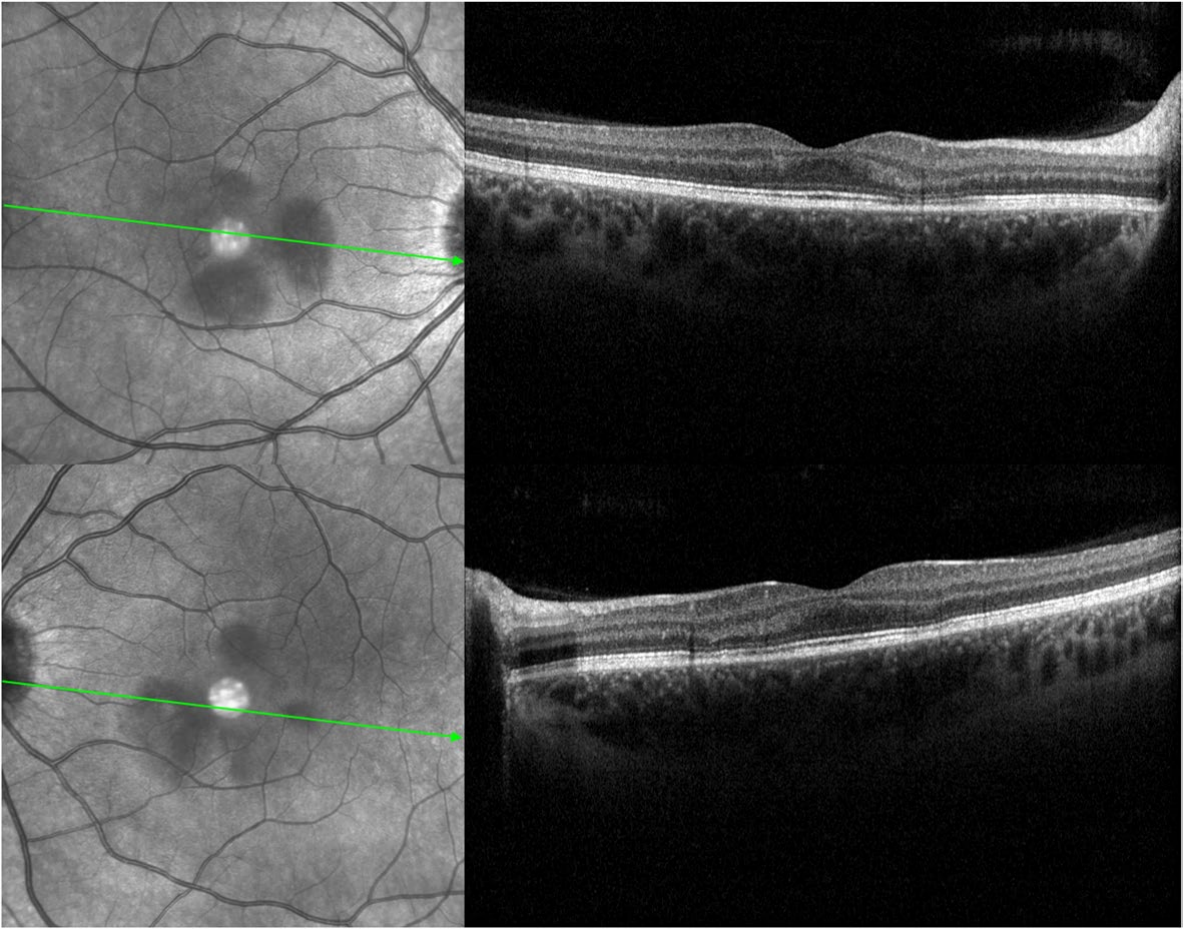
Infrared reflectance imaging and optical coherence tomography of both eyes. *Top Left*: On presentation, the near-infrared reflectance image of the right eye shows tear-shaped, hypo-reflective macular lesions. *Top Right*: The respective cross-sectional image displays slight hyperreflectivity of the outer plexiform layers, thinning of the outer nuclear layer, attenuation of the ellipsoid zone and disruption of interdigitation zone corresponding to one of the lesions. *Bottom Left*: the NIR image of the left eye shows tear-shaped, hypo-reflective perifoveal lesions. *Bottom Right*: The respective cross-sectional image of the left eye displays slight hyperreflectivity of the outer nuclear and plexiform layers, attenuation of the ellipsoid zone and disruption of interdigitation zone corresponding to the lesions

A 23-year-old Caucasian female with a sudden bilateral loss of vision with central scintillating scotomas. Her symptoms began 1 week after the first dose of COVID-19 vaccination. Her past medical history was unremarkable, with no other concomitant medications prescribed. No significant previous viral or pseudoviral episode was noticed. The best corrected visual acuity was 20/20 in both eyes. Fundus examination revealed in both eyes the classic wedge-shaped lesions with petaloid configuration and reddish-brown color (Fig. 1). Near-infrared reflectance (NIR) showed well-demarcated, dark grey lesions around the fovea. Spectral-domain optical coherence tomography (SD-OCT, Heidelberg, Germany) of the macula revealed hyperreflectivity within the outer plexiform layer, thinning of the outer nuclear layer and disruption of the ellipsoid and interdigitation zones, corresponding to the regions of NIR abnormalities (Fig. 2). These findings were compatible with the diagnosis of AMN.

**Fig. 3:**
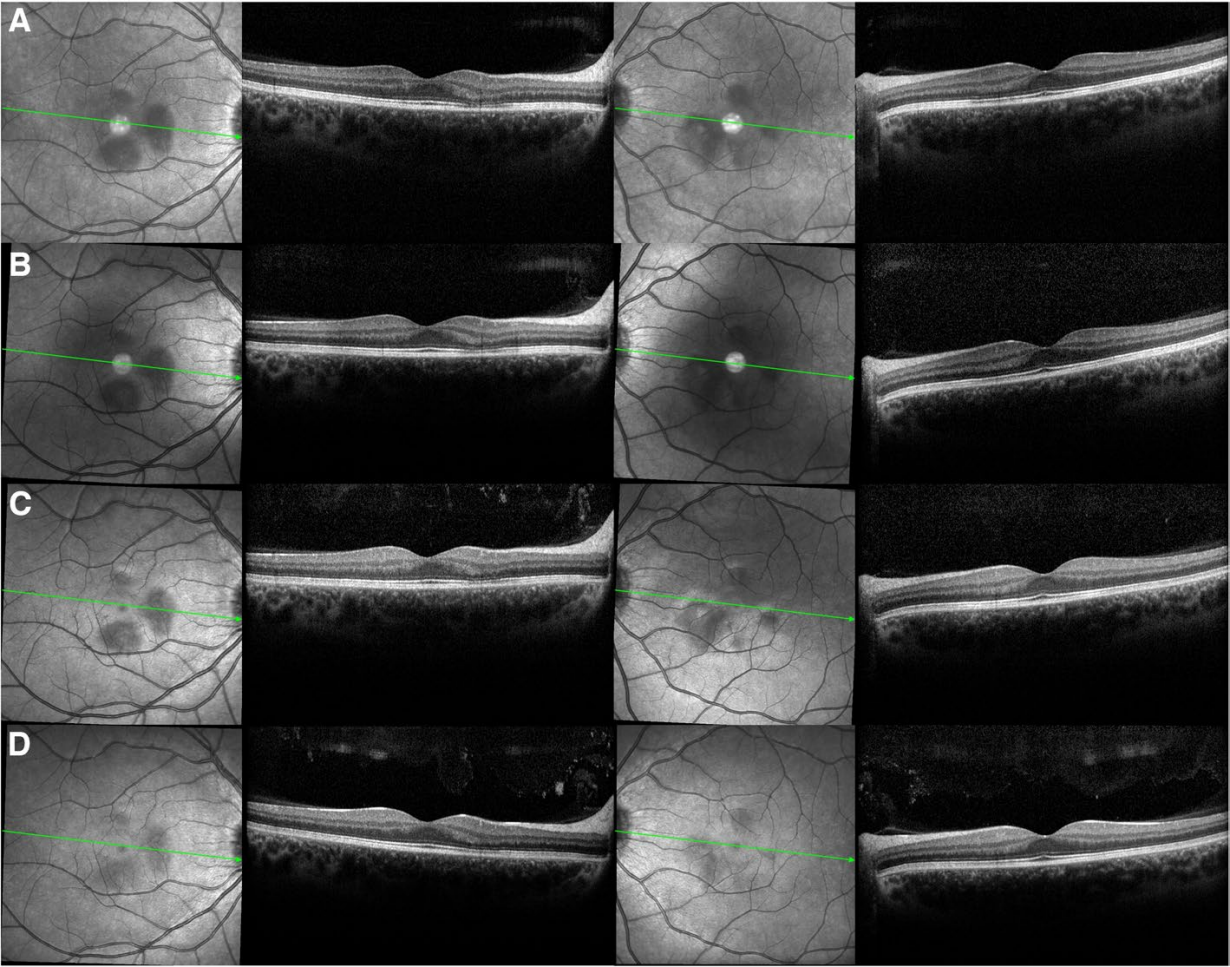
Sequential structural optical coherence tomography of both eyes. The figure illustrates the subsequent structural evolution of the original lesions after 10 days (**A**), 4 weeks (**B**), 12 weeks (**C**) and 24 weeks (**D**) of evolution, showing progressive fading of the infrared reflectance cuneiform lesions altogether with progressive resolution of the acute structural changes, leading to outer nuclear layer atrophy in both eyes

Throughout the subsequent follow-up, the patient progressively improved her visual field defects and the typical structural changes were fading away leaving a legacy of atrophy predominantly located at the level of the outer nuclear layer (Fig. 3). Her best corrected visual acuity remained 20/20 from baseline. Thereafter, the patient was lost to follow-up.

## Discussion

COVID-19 vaccines are categorized as viral vector vaccines, messenger RNA (mRNA) vaccines, inactivated vaccines, attenuated vaccines, and protein-adjuvant vaccines. The mRNA vaccines, such as Moderna and Pfizer/BioNTech vaccines, utilize RNA as the genetic material, encoding a specific viral protein. Consequently, these vaccines offer advantages such as not inducing immune responses specific to vectors, being non-infectious, and not integrating into the host genome. A few cases of Acute Macular Neuroretinopathy (AMN) have been reported after vaccination with the Moderna vaccine, while some cases have also been observed after BNT162b2 (BioNTech/Pfizer, Mainz, Germany) vaccination, but the majority have been associated with vector virus vaccines (AstraZeneca) [4–6].

The current hypothesis regarding the pathophysiology of AMN revolves around the occlusive vascular theory. A focal decrease in blood flow within the deep capillary plexus has been observed at the sites of the lesions, accompanied by a diffuse involvement of the choroid [7]. The distribution pattern of these lesions aligns with the anatomical structure of the lobules of the choriocapillaris. Since photoreceptors receive blood flow from both the choroidal and retinal circulations, their damage may lead to a reduced demand for oxygen and blood flow from both circulations. However, it remains unknown which circulation is primarily affected. In the case we report, optical coherence tomography angiography was not available to perform the examination.

Vaccine-induced immune thrombotic thrombocytopenia has not been associated with SARS-CoV-2 mRNA vaccinations [8]. Nonetheless, there have been suggestions of cardiac adverse events such as myocarditis or pericarditis [9]. Inflammation could play a significant role within the occlusive vascular component.

How could the Moderna vaccine potentially explain this vascular deficit? Infectious processes like influenza or vaccination stimulate the production of a substantial amount of type I interferon. This production is higher in women than in men and in younger individuals compared to older individuals [10]. Interferon is one of the cytokines that activate acute phase proteins, some of which have the potential to cause damage to the vascular endothelium [11, 12]. Furthermore, there might be a possible effect of oral contraceptives on the macula and the choroid, increasing susceptibility to vascular damage [13].

The cases described following COVID-19 vaccines exhibit typical characteristics in terms of sex, age, and risk factors, consistent with the usual presentation of AMN. However, the clear temporal sequence suggests a potential association, which may be dependent on the type of vaccine but could also be non-specific to any inflammatory response in susceptible individuals.

## Conclusions

It is estimated that more than 11 billion doses of COVID-19 vaccine have been administered globally. Despite this, AMN remains a rare pathology. But, if there is indeed a causal relationship, knowledge of the SARS-CoV-2 virus and its vaccines may help to elucidate the pathogenesis of AMN.

## Data Availability

Not applicable.
